# Ecological Risk and Human Health Implications of Heavy Metals Contamination of Surface Soil in E-Waste Recycling Sites in Douala, Cameroun

**DOI:** 10.5696/2156-9614-9.21.190310

**Published:** 2019-03-14

**Authors:** Romaric Emmanuel Ouabo, Mary B. Ogundiran, Abimbola Y. Sangodoyin, Babafemi A. Babalola

**Affiliations:** 1 Life and Earth Sciences Institute, Environmental Management, Pan African University, University of Ibadan, Nigeria.; 2 Department of Chemistry, Faculty of Science, University of Ibadan, Ibadan, Nigeria.; 3 Department of Agricultural and Environmental Engineering, Faculty of Technology, University of Ibadan, Ibadan, Nigeria.

**Keywords:** e-waste, Cameroon, heavy metals, ecological risk assessment, health risk

## Abstract

**Background.:**

Recycling of electronic waste (e-waste) in developing nations poses a risk to the environment and human health through the release of heavy metals.

**Objectives.:**

The aim of the present study was to evaluate the levels of heavy metals in Douala, Cameroun, the site of e-waste recycling activities.

**Methods.:**

Soil samples were collected from Makea, Ngodi and New Bell e-waste recycling sites, as well as from a control site. Samples were digested and levels of heavy metals were determined.

**Results.:**

The concentrations of the heavy metals in Makea occurred in the order of lead (Pb) (290±40) > zinc (Zn) (160±30) > chromium (Cr) (130±40) > copper (Cu) (130±20) > nickel (Ni) (56±5.7) > cadmium (Cd) (20±3.0); Pb (310±30) >Zn (150±20) >Cu (80±30) >Cr (70±40) >Ni (50±1.0) >Cd (30±5.0) in Ngodi; and Pb (280±40) >Zn (155±35) >Cu (80±50) >Cr (70±40) >Ni (53±2.0) >Cd (20±10) in New Bell. The levels of metals in all of the samples were higher compared to the control site, which was composed of vegetation and far from the e-waste sites, and in some cases, higher than permissible limits or guidelines. The ecological risk index of heavy metals for soil samples in all the e-waste sites indicated a very high risk.

**Conclusions.:**

Heavy metals concentrations in soil around e-waste recycling sites present serious health risks and further investigations are needed.

**Competing Interests.:**

The authors declare no competing financial interests.

## Introduction

Due to rapid development in the 20th century, the information and communication sector is the largest and fastest growing manufacturing industry in developing nations and throughout the world.^1^ Rapid growth in technology has led to frequent upgrades of electronic products and a high rate of discarded obsolete products, and this has resulted in a fast growing stream of municipal solid waste in the industrialized world over the last decade.^2–4^ Electronic waste (e-waste) is comprised of discarded electronic/electrical equipment such as computers, cellular phones, stereos, refrigerators, air conditioners and other consumer durables.

Approximately 41.8 million tons of e-waste was generated globally in 2014, out of which 6.5 million was handled by the national electronic take-back systems.^5^ In order to manage e-waste, many developed countries export approximately 50% - 80% of this e-waste to developing countries (e.g., China, India, Africa) for recycling and disposal due to lower labor costs and less stringent environmental regulations.^2^,^3^ According to the Basel Action Network, a Seattle-based environmental group, an estimated 500 shipping containers with a load equal in volume to 400,000 computer monitors or 175,000 large TV sets enter Lagos, Nigeria each month and 75% of such shipments are categorized as e-waste.^6^

E-waste is not hazardous waste by itself, but some components of e-waste can be harmful.^7^ E-waste may be comprised of rubber, glass, ceramics, ferrous and non-ferrous metals, plastics, printed circuit boards and other items. Iron and steel make up a large quantity of e-waste, followed by plastics, non-ferrous metals and other components.^8^ Precious metals like silver, gold, platinum and palladium are present in small quantities in equipment. E-wastes are hazardous in nature as a result of the presence of elements such as cadmium (Cd), mercury (Hg), arsenic (As), chromium (Cr), lead (Pb), selenium (Se) and flame retardants, etc., which are used in circuit boards, electrical wiring and computer batteries.^9–14^ The disposal of e-waste has led to a serious environmental problem, especially in developing countries, where many of the toxic components are released into different environmental media due to lack of proper management.^15^,^16^ Unregulated processing to recover precious metals such as open burning, melting, acid chemical bath and open dumping of unsalvageable materials can result in highly toxic pollution of heavy metals and other pollutants in aquatic and terrestrial ecosystems, as well as to the atmosphere.^17^,^18^

Heavy metals are of great concern due to their toxicity, mobility and non-biodegradability in environmental media such as soil, water and air. For example, heavy metals present in soil can be washed away by rainfall and end up in water bodies in the environment; they can contaminate groundwater through leaching, especially under acidic conditions.^5^,^19^,^20^ Since local residents usually rely on surface water and groundwater for irrigation and drinking, respectively, ecological risk assessment of heavy metals in the vicinity of e-waste recycling sites is needed.

Humans are exposed to heavy metals in soil through several routes, such as ingestion, inhalation and dermal absorption. It has been estimated that adults may ingest up to 100 mg dust/day.^21^ Children are usually exposed to greater amounts of dust than adults as a result of pica and play behavior.^22^ Exposure to high levels of heavy metals can lead to acute and chronic health effects, such as damage to central and peripheral nervous systems, blood, lungs, kidneys, liver and even death.^21^ Based on recent studies in China, elevated amounts of heavy metals and persistent toxic substances have been found in the blood of children and workers at e-waste recycling sites.^23^,^24^

**Figure 1:**
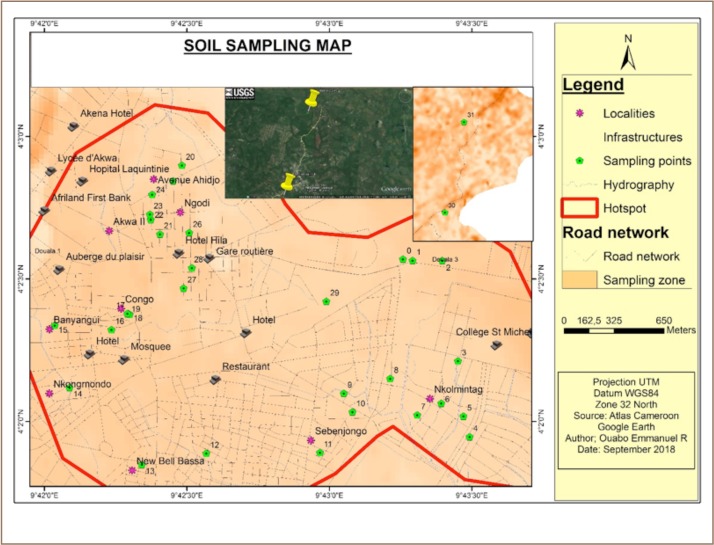
Map of sample location in Douala, Cameroon

The objectives of the present study were to investigate the levels of heavy metals present in surface soils from recycling sites in Douala, Cameroon and to estimate the potential ecological effects and health risks to adults and children.

## Methods

Makea, Ngodi and New Bell e-waste recycling sites are located in Douala, Cameroun. Makea has a population of 261,407 inhabitants, with 356,951 hectares of occupied space. The primary economic activity that takes place is informal e-waste recycling. Ngodi is approximately 265,102 hectares, with minor economic activities such as waste recycling and produce markets. There is a permanent population of 10,440 people, living directly within the area where e-waste activities take place daily. New Bell has a population of 55,587 inhabitants, with minimal economic activities such as e-waste recycling and produce markets. The area affected by e-waste site activities is approximately 233,937 hectares.

### Sampling

Makea, Ngodi and New Bell are the sites with the highest concentration of e-waste recycling activities in Douala. These three sites were chosen because of the population at risk and the intensity of activities, including open burning and dismantling of e-waste. Ten composite samples were collected from each site. Sampling was done between February and June 2017 and followed the method of the United States Environmental Protection Agency (USEPA).^25^ Surface soil samples (0–20 cm depth) were collected randomly from three informal e-waste recycling sites (Ngodi, Makea and New Bell) and a control site in Douala, Cameroun using manual coring. At each sampling site, composite samples were collected in clean polyethylene bags. Samples were stored in ice-filled coolers and transported to the laboratory. The soil samples were dried and sieved to <2 mm particle size.

### Sample digestion and analysis

Approximately 1.0 g of the soil sample was transferred into 50 mL digestion tubes, then 10 mL of 2 M nitric acid was added and samples were digested for 2 hours and shaken at 20 minute intervals.^26^ The digested samples were filtered into 25 mL flasks. The filtrates were diluted to the 25 ml mark with deionized water and stored in polyethylene bottles prior to instrumental analysis. The digested samples were analyzed for lead (Pb), nickel (Ni), zinc (Zn), cadmium Cd, chromium (Cr), copper (Cu) and cobalt (Co) using an atomic absorption spectrophotometer (AAS) (Buck scientific VGP 2010). Blank determination was also carried out without soil samples. The digested samples were analyzed in duplicates for quality control.

### Statistical analysis

Descriptive statistics such as mean, standard deviation and coefficient of variation of heavy metals were determined using Statistical Package for the Social Sciences (SPSS) software, version 17.0 (IBM SPSS Inc., Chicago, USA).

### Contamination factor and degree

In order to determine the contamination factor and degree of contamination in e-waste recycling sites, the formula of Thomilson et al. was used.^27^ The formulas for contamination factor and degree of contamination are as follows:

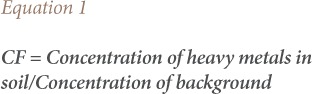


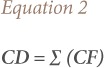
Where CF is the contamination factor and CD is the degree of contamination.

### Ecological risk assessment and potential ecological risk factor

The purpose of the ecological risk assessment is to assess the ecological effects of human activities in order to protect the environment. The assessment of the ecological risks of heavy metals in the soil samples was done using an ecological risk assessment and risk index proposed by Hakanson and reported in Huang et al.^28^,^29^ Environmental gradings by potential ecological risk index are presented in Table 1. The ecological risk factor quantitatively expresses the potential ecological risk of a given contaminant using the following equation:

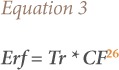
Where Erf is the ecological risk factor, Tr is the toxic-response factor for a given substance (the toxic-response factors for Pb, Ni, Zn, Cd, Cr and Cu are 5, 5, 1, 30, 2 and 5, respectively), and CF is the contamination factor.^28^

**Table 1:** Environmental Grading by Potential Ecological Risk Index^28^

Er value	Ecological risk grading of single metal	Risk index	Grade of potential ecological risk in the environment
Er< 1	Low risk	RI < 30	Low risk
5 - 9	Moderate risk	30 - 59	Moderate risk
10 - 19	Considerable risk	60 - 120	Considerable risk
20 - 40	High risk	RI ≥ 120	Very high risk
Er ≥ 40	Very high risk		

Abbreviations: Er, ecological risk; RI, risk index.

### Human health risk assessment

A health risk assessment is carried out to estimate the type and magnitude of the exposure compared to the chemical elements present in soil. According to the USEPA methodology, risks for metal exposure are calculated by estimating direct exposure to soil.^30^,^31^ Three pathways are considered: (i) incidental ingestion of soil, (ii) inhalation of particulates emitted from soil, and (iii) dermal contact with soil. The chronic daily intakes (CDI) through the three pathways were estimated with Equations 4–6:

**A. Ingestion of soil**

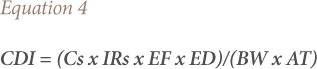

**B. Dermal contact with soil**

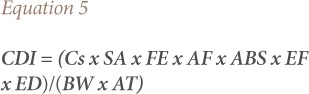

**C. Inhalation of particulates emitted from soil**

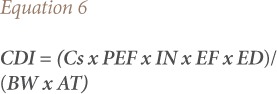

Where Cs is concentration of the heavy metal in soil, IRs is the ingestion rate in soil, SA is the exposed skin surface area, FE is the dermal exposure ratio, AF is the soil to skin adherence factor, ABS is the dermal absorption factor, PEF is the particulate emission factor, IN is the inhalation rate, EF is the exposure frequency, ED is the exposure duration, BW is body weight and AT is the average time of exposure. The values for the parameters are given in Table 2.

**Table 2:** Exposure Parameters Used for the Health Risk Assessment Through Different Exposure Pathways for Soil^31^,^32^

**Parameter**	**Unit**	**Child**	**Adult**
Body weight (BW)	kg	15	70
Exposure frequency (EF)	days/year	250	250
Exposure duration (ED)	years	6	30
Ingestion rate (IR)	mg/day	200	100
Inhalation rate (IN)	m^3^/day	10	20
Skin surface area (SA)	cm^2^	5800	20150
Soil adherence factor (AF)	mg/cm^2^	0.2	0.07
Dermal absorption factor (ABS)	none	0.1	0.1
Dermal exposure ratio (FE)	none	0.61	0.61
Particulate emission factor (PEF)	m^3^/kg	1.3 × 10^9^	1.3 × 10^9^
Conversion factor	kg/mg	10^−6^	10^−6^
Average time (AT)	days		
For non-carcinogens		365 × ED	365 × ED

The basic equation for calculating systemic toxicity or non-carcinogenic hazard for a single substance/element is expressed as the hazard quotient:

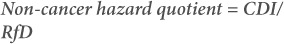
Where the non-cancer hazard quotient is a unitless number that is expressed as the probability of an individual suffering an adverse effect, CDI is the sum of the chronic daily intake of a toxicant expressed in mg/kg/day from different pathways, that is, soil, water, dermal and air, and RfD is the chronic reference dose for the toxicant expressed in mg/kg/day.

As a rule, the greater the value of CDI/RfD above unity, the greater the level of concern. The values for reference doses for different elements are shown in Table 3.

**Table 3:** Reference Doses for Heavy Metals

Heavy metal	Oral Rfd	Dermal Rfd	Inhalation Rfd	Reference
Pb	3.60E-03	-	-	31
Cd	5.00E-04	5.00E-04	5.70E-05	31
Ni	2.00E-02	5.60E-03	-	31
Cu	3.70E-02	2.40E-02	-	31
Zn	3.00E-01	7.50E-02	-	31

All units expressed in mg/kg-day. Abbreviations: RfD, reference doses.

## Results

**Figure 2:**
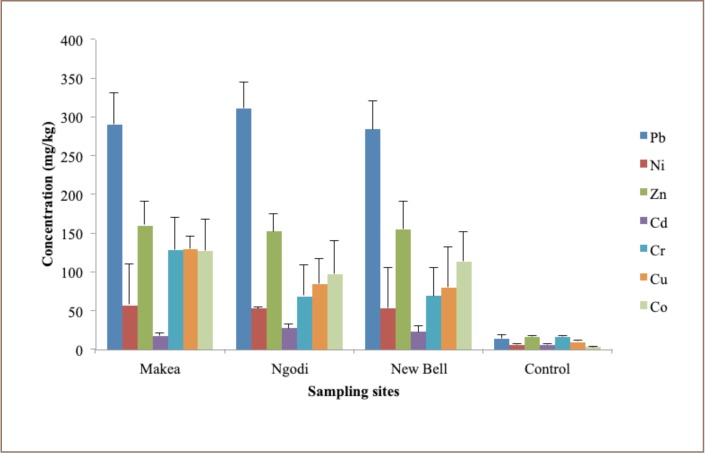
Mean concentrations of heavy metals in the soil of e-waste management sites compared with the control site

The concentrations of the heavy metals in soil samples at the Makea, New Bell and Ngodi e-waste processing and control sites are shown in Table 4. The levels of Zn and Cr in the e-waste processing sites were higher than the control site, but lower than the standard guideline values for different countries, including Europe and China. However, the levels of Pb, Ni, Cd and Cu were higher than the control site and the standard values for different countries including Europe and China's standard guideline value for Pb or Cd.^33^,^34^ The mean concentrations of heavy metals in the soil of the e-waste management sites compared with the control site are shown in Figure 2.

**Table 4:** Heavy Metal Concentrations in Soil from E-Waste Sites in Douala, Cameroun

**Sites**	**N**		**Heavy metals (mg/kg)**					
			Pb	Ni	Zn	Cd	Cr	Cu
**Makea**	10	Mean	290±40	56±5.7	160±30	20±3.0	130±40	130±20
		Range	210–340	52.0–60	100–210	10–20	80–200	110–170
**Ngodi**	10	Mean	310±30	50±1.0	150±20	30±5.0	70±40	80±30
		Range	260–370	50.0–54.0	100–200	20–30	10–130	10–120
**New Bell**	10	Mean	280±40	53.0±2	155±35	20±10	70±40	80±50
		Range	230–340	50–60	100–220	10–30	20–140	20–180
**Control**	2	Mean	10±4.0	10±0.0	20±0.0	6.0±0.0	20±0.4	10±1
		Range	10–20	10–10	20–20	10–10	20–20	10–10
**Europe**			300	75	300	3	150	140
**China**			80	50	250	0.5	200	100

### Analysis of variance of heavy metals in soil

There was significant variation in the concentration of the heavy metals in the soil samples collected in e-waste sites in Douala (Makea, Ngodi, and New Bell), based on the one-way analysis of variance (ANOVA), as shown in Table 5.

**Table 5:** Analysis of Variance of Heavy Metals in Soils

		Sum of Squares	df	Mean Square	F	p-value
Pb	Between groups Within groups Total	544744.892 76868.801 621613.693	7 56 63	77820.699 1372.657	56.693	.000
Ni	Between groups Within groups Total	14228.645 1452.609 15681.254	7 56 63	2032.664 25.939	78.362	.000
Zn	Between groups Within groups Total	58772.728 48199.930 106972.658	7 56 63	8396.104 860.713	9.755	.000
Cd	Between groups Within groups Total	5032.837 1266.016 6298.853	7 56 63	718.977 22.607	31.803	.000
Cr	Between groups Within groups Total	166452.594 62561.945 229014.539	7 56 63	23778.942 1117.178	21.285	.000
Cu	Between groups Within groups Total	65927.990 60427.975 126355.965	7 56 63	9418.284 1079.071	8.728	.000

Abbreviations: df, degree of freedom; F, F-test

### Ecological risk assessment and risk index of heavy metals in soil

The results of the ecological risk assessment and risk index of heavy metals in the soils are shown in Table 6. Across all of the e-waste sites (Makea, Ngodi and New Bell), the ecological risks of Pb, Ni, Cu and Cd were very high. The Cr risk was considerable across all of the e-waste sites, but Zn evidenced a very low risk. Altogether, the computed risk index of the three sites showed a high level of risk.

**Table 6:** Ecological Risk Assessment and Risk Index of Heavy Metals in Soil from E-Waste Sites in Douala

Heavy metals	Ecological Risk		

	Ngodi	Makea	New Bell
Pb	223	220	215
Ni	83.5	100	93.0
Zn	3.12	3.24	3.35
Cd	269	238	247
Cr	14.3	18.4	14.4
Cu	101	126	103

**Risk Index**	**840**	**854**	**833**

### Non-carcinogenic risk of heavy metals for adults and children

**Figure 3:**
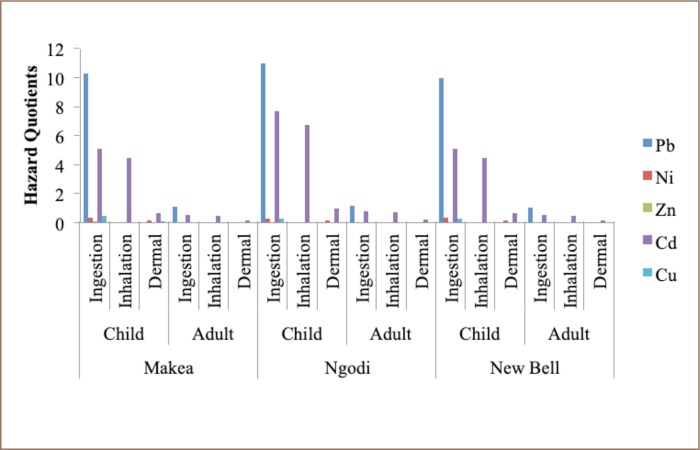
Hazard quotient values for non-carcinogenic heavy metals in adults and children for soil from e-waste recycling sites in Douala, Cameroun

The non-carcinogenic risk for adults and children in Ngodi, Makea and New Bell was calculated and the average daily intake values are presented Table 7. The results for ingestion, inhalation and dermal pathways are all presented in terms of hazard quotient, as shown in Figure 3. In Makea, Ngodi and New Bell, children had no cancer risk for Cd, unlike adults.

**Table 7:** Average Daily Intake of Heavy Metals for Adults and Children in Soil from the E-Waste Recycling Sites in Douala for Non-Carcinogenic Risk Calculations

		Pathway	Average daily intake (mg/kg/day)								

			**Pb**	**Ni**	**Zn**	**Cd**	**Cu**	**Total**

Makea	Child	ADI ing	3.71E-02	7.16E-03	2.05E-02	2.56E-03	1.66E-02	8.39E-02
		ADI inh	3.71E-03	7.16E-04	2.05E-03	2.56E-04	1.66E-03	8.39E-03
		ADI derm	4.75E-03	9.17E-04	2.62E-03	3.28E-04	2.13E-03	1.07E-02
		**Total**	**4.44E-02**	**1.06E-02**	**2.56E-02**	**5.44E-03**	**2.13E-02**	**1.07E-01**
	Adult	ADI ing	3.97E-03	7.67E-04	2.19E-03	2.74E-04	1.78E-03	8.99E-03
		ADI inh	3.97E-04	7.67E-05	2.19E-04	2.74E-05	1.78E-04	8.99E-04
		ADI derm	9.84E-04	1.90E-04	5.43E-04	6.79E-05	4.41E-04	2.23E-03
		**Total**	**5.35E-03**	**1.03E-03**	**2.95E-03**	**3.69E-04**	**2.40E-03**	**1.21E-02**
Ngodi	Child	ADI ing	3.96E-02	6.39E-03	1.92E-02	3.84E-03	1.02E-02	7.93E-02
		ADI inh	3.96E-03	6.39E-04	1.92E-03	3.84E-04	1.02E-03	7.93E-03
		ADI derm	5.08E-03	8.19E-04	2.46E-03	4.9 IE-04	1.31E-03	1.02E-02
		**Total**	**4.73E-02**	**9.77E-03**	**2.42E-02**	**6.88E-03**	**1.41E-02**	**1.02E-01**
	Adult	ADI ing	4.25E-03	6.85E-04	2.05E-03	4.11E-04	1.10E-03	8.49E-03
		ADI inh	4.25E-04	6.85E-05	2.05E-04	4.11E-05	1.10E-04	8.49E-04
		ADI derm	1.05E-03	1.70E-04	5.09E-04	1.02E-04	2.71E-04	2.10E-03
		**Total**	**5.72E-03**	**9.23E-04**	**2.77E-03**	**5.54E-04**	**1.48E-03**	**1.14E-02**
New Bell	Child	ADI ing	3.58E-02	6.78E-03	1.98E-02	2.56E-03	1.02E-02	7.52E-02
		ADI inh	3.58E-03	6.78E-04	1.98E-03	2.56E-04	1.02E-03	7.52E-03
		ADI derm	4.59E-03	8.68E-04	2.54E-03	3.28E-04	1.31E-03	9.63E-03
		**Total**	**4.29E-02**	**1.02E-02**	**2.49E-02**	**5.44E-03**	**1.41E-02**	**9.76E-02**
	Adult	ADI ing	3.84E-03	7.26E-04	2.12E-03	2.74E-04	1.10E-03	8.05E-03
		ADI inh	3.84E-04	7.26E-05	2.12E-04	2.74E-05	1.10E-04	8.05E-04
		ADI derm	9.50E-04	1.80E-04	5.26E-04	6.79E-05	2.71E-04	1.99E-03
		**Total**	**5.17E-03**	**9.78E-04**	**2.86E-03**	**3.69E-04**	**1.48E-03**	**1.09E-02**

Abbreviations: ADI, average daily intake; ADI derm, average daily dose through dermal contact; ADI ing, average daily dose thorugh ingestion; ADI inh, average daily dose through inhalation; E, exponential

## Discussion

The concentrations of Pb at the three study sites were higher than at the control site and greater than permissible limits in China. Elevated mean levels of Pb in soils may eventually migrate into human systems and produce toxic effects on various systems in the body, such as the central and peripheral nervous systems, genitourinary system and reproductive system.^35^ Elevated Pb levels at the e-waste site could have resulted from burning of e-waste, such as refrigerators, computers, cables, batteries and air conditioners, among others.^36^ The mean concentrations of Ni in the soil at Makea, Ngodi and New Bell were higher than the concentration at the control site and the permissible limits in Europe and China.

The mean concentrations of Zn in the soil at Makea, Ngodi and New Bell were higher than the concentration at the control site and lower than the permissible limits in Europe and China. Zinc is an important nutritional element, but is harmful in high quantities. Large amounts of Zn can cause metal fume fever.^21^ The mean concentrations of Cd in soil at Makea, Ngodi and New Bell were higher than the concentration in the control site and the permissible limits in Europe and China. Cadmium can accumulate in the human body, especially in the kidneys. Cadmium has been associated with progressive renal tubular dysfunction.^37^ The mean concentrations of Cr in the soil at Makea, Ngodi and New Bell were higher than the concentration in the control site. Toxicity of Cr is dependent on its oxidation state. When it exists in hexavalent form, it can cause disease, such as skin rashes, kidney and liver damage and cancer.^38^ The mean concentrations of Cu in soil at Makea, Ngodi and New Bell were higher than the concentration in control site and the permissible limits in Europe and China.

It is clear that the heavy metals contamination at the e-waste recycling sites was the result of improper disposal methods. The concentrations of metals were higher than the permissible limits set for environmental quality standards for soils in Europe and China. Open dumping and dismantling are the most common methods of handling e-waste in recycling sites in Douala. This is due to weak laws and regulations against open burning or dismantling of e-waste and the lack of an e-waste management system. Ashes produced by open-burning sites require careful management because they are a source of heavy metal contamination.

### Ecological risk assessment and risk index of the elements in soil

The ecological risk of heavy metals for soil samples in all of the e-waste sites indicated that Pb, Ni, Cd and Cu could pose a very high risk. Zinc was of low risk and Cr was of considerable risk. The risk index of heavy metals for the soils across all e-waste sites suggested a very high risk for these metals in the environment.

### Non-carcinogenic risk of heavy metals for adults and children

The heavy metals used to calculate the non-carcinogenic risk included Pb, Ni, Zn, Cd and Cu. It has been established that the non-cancer risk is a hazard quotient ≥ 1. In Makea, Ngodi and New Bell, children and adults are at risk of Pb toxicity due to the large amounts of Pb found, most likely through ingestion pathways. As a result of the large amounts of Cd present in e-waste sites in Makea, New Bell and Ngodi, children are exposed to Cd toxicity through the ingestion and inhalation pathways. Therefore, the hazard coefficient indicates a high risk of health issues such as cancer, kidney and lung damage and high blood pressure. The hazard index for children decreased across the sites in the order of Ngodi> Makea>New Bell. The hazard index for adults increased across the sites in the order of New Bell< Makea< Ngodi. It is therefore recommended that further studies be carried out at these e-waste recycling sites in order to limit the adverse impacts of heavy metals contamination and facilitate the application of remediation measures.

## Conclusions

Soil contamination by heavy metals due to e-waste recycling processes in Douala, Cameroun was observed. The uncontrolled processing of e-waste in Douala has likely resulted in the release of elevated levels of elements into the surrounding soil. The levels of heavy metals in soil were higher at the recycling sites compared to the control site and international standards. The ecological risk at all of the sites was high. The hazard quotient results also revealed that adults and children near the e-waste recycling sites are at risk of Pb and Cr toxicity through the ingestion and inhalation pathways. Findings from the present study demonstrate that urgent measures are needed in order to reduce heavy metals contamination resulting from e-waste recycling activities in Douala, Cameroun.
